# Bilateral tinnitus associated with a diffuse astrocytoma of Heschl’s gyrus: a case report and review of literature

**DOI:** 10.1093/jscr/rjae317

**Published:** 2024-05-18

**Authors:** Rabii Aboulhosn, Peter Bodkin

**Affiliations:** School of Medicine, University of Aberdeen, Forester hill, Aberdeen AB25 2ZD, United Kingdom; Department of Neurosurgery, Aberdeen Royal Infirmary, Aberdeen AB25 2ZN, United Kingdom; Department of Neurosurgery, Aberdeen Royal Infirmary, Aberdeen AB25 2ZN, United Kingdom

**Keywords:** tinnitus, Heschl’s gyrus, diffuse astrocytoma, neurosurgery

## Abstract

Auditory processing is initiated within the primary auditory cortex, concealed within the sylvian fissure bilaterally on a collection of gyri described as Heschl’s Gyrus (HG). Glial neoplasms localized to or involving HG are rare. The main symptoms of these tumours are complex partial seizures characterized by auditory features. Here, we describe an unusual case of bilateral tinnitus and hemi-paraesthesia associated with a HG diffuse astrocytoma. Bilateral tinnitus secondary to intrinsic brain tumours is atypical. Bilateral tinnitus is frequently observed in patients with noise-induced hearing loss, presbycusis, ototoxic medication, and metabolic and psychiatric disease. In the case we present, the synchronous sensory and auditory symptoms are likely due to seizure activity affecting the primary auditory and somatosensory cortex. In a patient presenting with chronic, bilateral tinnitus with no known underlying otologic disease which is associated with hemi-body paraesthesia, we would advocate for consideration of brain imaging to exclude pathology in HG.

## Introduction

Auditory processing is initiated within the primary auditory cortex (PAC), concealed within the Sylvian fissure bilaterally on a collection of gyri described as Heschl’s Gyrus (HG). The PAC occupies the posteromedial two-thirds of HG, while plan temporale and superior temporal gyrus constitute areas of the secondary auditory association cortex [1, 2]. Functional magnetic resonance imaging (MRI) studies have revealed that neurological activity within HG contributes to the development of tinnitus, which describes the perception of a continuous or intermittent sound in the absence of a corresponding external acoustic stimulus [3].

Intrinsic brain tumours localized to or involving HG are rare. The main symptoms of these lesions are complex partial seizures, characterized by auditory features, with or without secondary generalization. While unilateral tinnitus has been described in this setting, bilateral tinnitus is less typical.

Here, we describe an unusual case of bilateral tinnitus and hemi-paraesthesia associated with a HG diffuse astrocytoma.

## Case report

A 32-year-old right-handed male, an apprentice mechanical engineer without any comorbidity, experienced an episode of concurrent bilateral tinnitus and left hemi-body paraesthesia involving the face, arm, and leg. He described the tinnitus as a ‘high pitched buzzing sound’ and the paraesthesia as ‘numbness’ which started in the face and expanded to the extremities. The patient denied any headache or associated confusion and there were no other otologic symptoms, olfactory perceptions, nausea, vomiting, limb weakness, hypoesthesia, photophobia, or phonophobia. The patient went to Accident and Emergency as he was concerned about a stroke, but they identified no deficit.

These episodes of synchronous bilateral tinnitus and left hemi-body paraesthesia continued to occur intermittently, on average two to three times a week, and were most pronounced in the morning. The episodes were sudden onset and offset, lasting a few seconds in duration. He continued to deny other otologic and neurological symptoms.

Neurological and cranial nerve examination were normal. The patient’s hearing tests were also normal.

**Figure 1:**
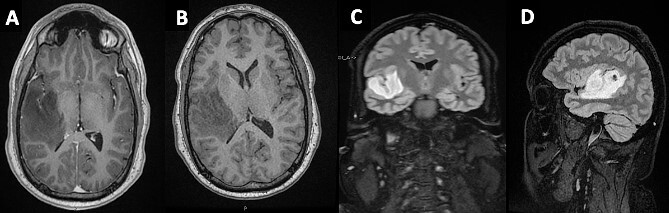
Mass lesion occupying the HG on the right temporal gyrus on (A) axial post-contrast T1 - (B) axial non-contrast T1- (C + D) T1-weighted sagittal and coronal FLAIR MRIs, respectively.

In light of the persistent synchronous symptoms, imaging was obtained. An MRI head revealed a non-enhancing, T1-hyperintense lesion within the HG in the right cerebral hemisphere (Fig. 1), extending into the insular cortex. A Stealth guided burrhole biopsy of the right temporal lobe was performed. Pathological analysis revealed a Grade II diffuse astrocytoma.

The patient received a course of 500 mg Levetiracetam which resolved the tinnitus and paraesthesia.

## Discussion

Tinnitus is characteristically described as buzzing, clicking, or pulsating [4]. It is a prevalent symptom, with a prevalence up to 37.2%, and has significant implications on quality of life [5]. Various metrics are used to classify tinnitus (Table 1).

**Table 1:** Tinnitus descriptions.

**Type of Tinnitus**	**Description**
Subjective	Tinnitus only experienced by affected individual
Objective	Observer can hear the sounds as well
Pulsatile	Sound corresponds with heart pulsations
Unilateral	Sound perceived only in one ear
Bilateral	Sound perceived in both ears
Primary	Idiopathic aetiology
Secondary	Underlying aetiology associated
Acute	Present for <6 months
Chronic	Present for >6 months
Peripheral	Abnormal neural activity at cochlear nerve level
Central	Abnormal neural activity in auditory centres

Population-based data indicate that noise-induced hearing loss represents the most prevalent risk factor for tinnitus [6, 7]. While risk factors have been identified, the pathophysiology of tinnitus remains elusive. A commonly referenced model suggests that tinnitus occurs in response to neuroplastic changes in central auditory structures secondary to cochlear pathology. In neoplastic disease, particularly cerebellopontine angle tumours, tinnitus may be observed due to the mass effect on the vestibulocochlear nerve [8, 9].

Glioma’s localized to or involving HG are rare, only limited to few case reports in the English literature. Russel and Golfinos describe two patients who experienced piercing and muffled sounds preceding episodes of generalized seizures [10]. Like our case, biopsy samples revealed a low-grade astrocytoma involving the HG. They also discuss another case of auditory episodes characterized by a loud piercing beep associated with an astrocytoma of HG. In another study, Silbergeld [11] describes a patient with a low-grade astrocytoma involving HG who experienced a generalized tonic–clonic seizure which was preceded by ringing in the contralateral ear. Similarly, Benzagmout *et al*. discuss a patient who experienced auditory seizures secondary to a glioma involving the PAC [12]. Like our case, the symptoms resolved with anticonvulsant medication. In other work, Yamamoto and colleagues report a patient with a superior temporal lobe anaplastic astrocytoma involving HG, which presented as generalized seizures and cognitive decline [13].

Bilateral tinnitus associated with lesions involving HG, as presented in this case, is unusual. Bilateral tinnitus is frequently observed in patients with noise-induced hearing loss, presbycusis, head trauma, ototoxic medication, and metabolic and psychiatric disease [8]. In the case we present, the synchronous sensory and auditory symptoms are likely due to seizure activity affecting the primary auditory and somatosensory cortex given the response to the anticonvulsant medication. Invasion of limbic structures, including the amygdala and hippocampus, may explain why insular and temporal LGG initially manifest as complex partial seizures [14]. However, it remains difficult to fully ascertain seizure activity as electroencephalography was not obtained. Tumour-induced hypoperfusion of the primary auditory and somatosensory cortex may also be contributing to the development of the symptoms.

## Conclusion

Lesions localized to or involving HG may present with auditory features, including bilateral tinnitus. In a patient presenting with chronic, bilateral tinnitus with no known underlying otologic disease which is associated with hemi-body paraesthesia, we would advocate for consideration of brain imaging to exclude pathology in HG.

## Conflict of interest statement

The authors declare no conflicts of interest.

## Funding

The authors received no financial support for this research.

## Informed consent

Written informed consent for the paper to be published was obtained from the patient.
